# Influencing Factors and Kinetics of Modified Shell Powder/La-Fe-TiO_2_ Photocatalytic Degradation of Pyridine Wastewater

**DOI:** 10.3390/ijerph192214835

**Published:** 2022-11-11

**Authors:** Jinkai Shu, Bozhi Ren, Wei Zhang, An Wang, Sen Lu, Shuyu Liu

**Affiliations:** 1 School of Municipal and Surveying Engineering, Hunan City University, Yiyang 413000, China; 2 School of Civil Engineering, Hunan University of Science and Technology, Xiangtan 411201, China; 3 Hunan Provincial Village Drinking Water Quality Safety Engineering Technology Research Center, Yiyang 413000, China; 4 Hunan Provincial Key Laboratory of Shale Gas Resource Exploitation, Xiangtan 411201, China

**Keywords:** La-Fe-TiO_2_@MSP, photocatalysis, pyridine, reaction kinetics

## Abstract

Modified Shell Powder/La-Fe-TiO_2_ (La-Fe-TiO_2_@MSP) composites were fabricated using the sol-gel method and characterized by SEM, XRD, UV-vis DRS and photocurrent techniques, and their physicochemical and optical properties were analyzed. The effects of various factors on the photocatalytic degradation of pyridine and its reaction kinetics were investigated by batch experiments using pyridine, a typical nitrogen-containing heterocyclic compound in coal chemical wastewater, as the target removal species. The pyridine degradation rate of 80.23% was obtained for 800 mg/L composite solution by photocatalytic oxidation of 50 mg/L pyridine wastewater for 180 min at 35 °C, pH = 8 and light intensity of 560 W. The photocatalytic degradation performance was optimal. The quenching experiments determined that the active species of photodegradation were mainly hole and hydroxyl radicals, and the photocatalytic degradation mechanism was analyzed in this way.

## 1. Introduction

Recently, rapid development of the coal chemical industry has given rise to the problem of “three highs”, namely, “high water consumption, high energy consumption and high pollution”, which have greatly restricted the sustainable development of societies. Shanxi is an important province in China in terms of coal transportation, but the inverse distribution of coal and water resources with regional ecological development has forced Shanxi province to shoulder the heavy burden of development on the one hand and bear the huge pressure of environmental pollution on the other hand [1,2]. With the continuous discharge of coal chemical wastewater, local water environments are rapidly deteriorating. Chemical wastewaters containing nitrogenous heterocyclic compounds such as pyridine, quinoline and indole as the main pollutants are produced in high content volumes. These wastewaters have poor biodegradability, and can present “teratogenic, carcinogenic, and mutagenic” effects [3,4,5]. Furthermore, they are easily mixed with water and alcohol in the environment, and once released into nature, they pose a clear threat to plants, animals and humans. Therefore, this type of organic wastewater needs to be treated before being discharged [6,7,8].

There are many kinds of coal chemical wastewater treatment processes, among which adsorption, photocatalytic oxidation and microwave catalysis are common. Adsorption has the advantages of fast adsorption, convenience and stability, without secondary pollution. Kang et al. developed an amino functionalized graphene oxide aerogel to adsorb quinoline with gigantic potential; after eight adsorption desorption cycles, about 89.54% of the original adsorption capacity was still retained [9]. Furthermore, Gao et al. studied the feasibility of coking coal adsorbing organic pollutants in coking wastewater, and the results showed that coking coal is a suitable low cost adsorbent for the removal of stubborn organic pollutants [10]. The adsorption method is not effective for the treatment of water-soluble substances, and the problems of recovery and regeneration have to be solved. At present, it is widely known that the research on adsorption materials mainly focuses on the improvement of traditional adsorption materials and the research of “green” adsorption materials [11,12]. It is of great significance to develop new adsorption materials suitable for the removal of nitrogen-containing heterocyclic compounds. Photocatalytic oxidation takes advantage of the strong oxidation capacity of the photocatalyst oxidant to open the ring and remove nitrogen, before finally realizing complete mineralization, making it lose toxicity without secondary pollution. Among them, TiO_2_ has become a research hotspot of photocatalysts due to its high chemical stability, non-toxic, low cost and other advantages, and its photostability and efficiency are highly favored [13,14,15,16,17]. Ortega Liébana et al. found that 3-chloropyridine was completely mineralized under TiO_2_ photocatalysis [18].

TiO_2_ photocatalysis can improve the biochemical properties of nitrogen-containing heterocyclic compounds such as pyridine in coking wastewater with a better degradation effect and has the very big superiority in treating coking wastewater; however, there are some problems, such as the easy compounding of electron–hole pairs and narrow spectral response range [19]. Doping with metal ions is a major way to modify TiO_2_. Studies have demonstrated that Fe^3+^ is an effective dopant ion. Fe^3+^ can capture the photogenerated electrons, thus reducing the recombination probability of photogenerated hole pairs and further enhancing the photocatalytic efficiency of TiO_2_ [20,21,22,23]. Rare earth element ion doping is another important approach to modify TiO_2_. The La atomic number of rare earth elements is 67, which has the most stable +3 valence, and the higher the possibility of generating ions, the higher the tendency of valence change, which makes the 4f electrons of other elements easy to generate electron leap by mating in the 4f orbitals. Its special electronic behavior allows the doping of rare earth particles La^3+^ to modify the photocatalytic properties and improve the photocatalytic activity of TiO_2_ [24,25,26,27,28,29]. Therefore, the co-doping of La-Fe ions can both improve the quantum yield and broaden the photo-response range of TiO_2_, optimizing the performance of photocatalytic material properties and improving the photocatalytic degradation efficiency.

TiO_2_-based composites have gradually become the focus of attention in the field of photocatalysis [30,31,32], and adsorbent materials can transfer typical nitrogen-containing heterocyclic compounds from aqueous phase to the solid phase, so the combination of suspended TiO_2_ and high efficiency adsorption materials can effectively improve the problems of easy loss of the suspended phase, difficult separation and recovery and easy loss of active ingredients, etc. Many scholars have conducted a series of studies on this type of composite photocatalytic material [32,33,34,35]. Currently, the preparation of new, inexpensive, “green” adsorbents from waste is a hot spot for research [36,37], and shell is a kind of aquatic organism with high yield and strong environmental adaptability, which absorbs elements from the surrounding environmental media and forms a shell with carbonate composition through the secretion of its soft body (rich in CaCO_3_ and a large number of micropores). Therefore, it is necessary to explore the loading of photocatalysts on modified shells to degrade the nitrogenous heterocyclic compounds transferred to this adsorbent into inorganic substances with relatively low molecular weight through a certain amount of hydroxyl radicals generated in water. Furthermore, it is necessary to reduce the effect of spatial site resistance and improve the removal effect to achieve the efficient and harmless treatment of nitrogenous heterocyclic compounds in water.

In this paper, rare earth element La and metal ion Fe were used to load TiO_2_ on the modified shell powder carrier to form a stationary phase. Pyridine-containing wastewater was used as the target degradation product. The effects of composite material dosage, solution pH, initial concentration and photocatalytic time on the degradation effect were investigated. The degradation kinetics were analyzed in order to provide the data support and technical reference for the degradation of nitrogen-containing heterocyclic compounds, and to provide a theoretical reference for the further in-depth treatment of coal chemical industry wastewater, so as to improve the pollution control technology of the coal chemical industry; resolve the contradiction between resources and the environment; realize the clean transformation of coal; promote the safe, green, innovative and sustainable development of the coal chemical industry; and strive to achieve “zero emission” of the coal chemical industry wastewater as soon as possible, in order to achieve carbon neutrality.

## 2. Materials and Methods

### 2.1. Materials

The experimentally employed pyridine wastewater was prepared using pyridine and pure water according to the desired concentration. Tributyl titanate, isopropyl alcohol, lanthanum nitrate, ferric nitrate, hydrochloric acid, sodium hydroxide and pyridine were of analytical grade and were purchased from the Aladdin industrial corporation. Deionized water was used throughout the experiments. The raw material used in experiments was natural shells, which were collected from Dongting Lake.

### 2.2. Procedures

A certain amount of modified shell powder was weighted and added into 60 mL isopropanol solution. Then, it was ultrasonicated for 8 min to evenly disperse the modified shell powder. Then, 15 mL tetrabutyl titanate was dropwise added under magnetic stirring to form solution A.

Certain amounts of lanthanum nitrate and iron nitrate crystals were added to a mixture of 20 mL isopropyl alcohol and 2.4 mL deionized water, and the pH of the solution about neutral was adjusted by hydrochloric acid to form solution B. Solution B was slowly added dropwise to solution A and sol was fabricated after magnetic stirring for 2 h. The mixture was aged at room temperature for 12 h, dried and ground and finally calcined in a muffle furnace to obtain the composite material for the subsequent photocatalytic experiments.

**Figure 1:**
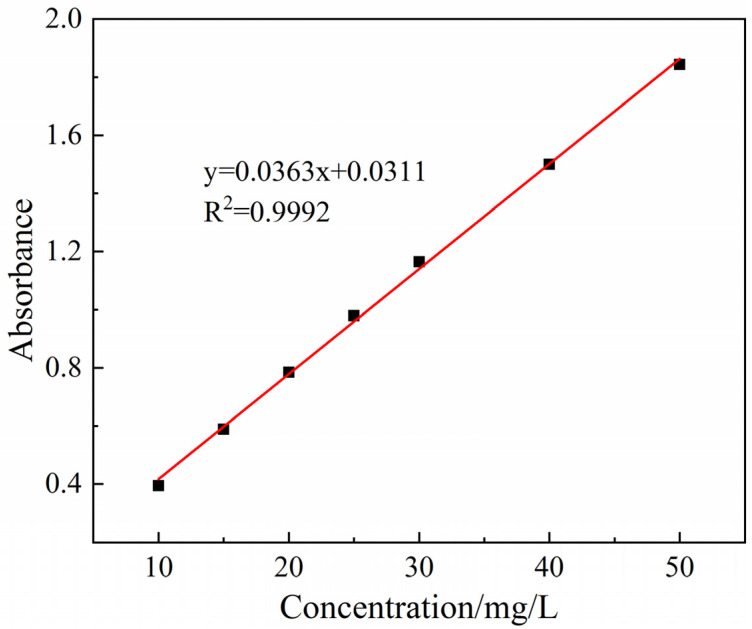
Standard curve of pyridine.

The absorbance magnitudes of pyridine solutions with different concentrations were measured by a 4802S UV-vis spectrophotometer at a maximum wavelength of 256 nm to plot standard curves, as Figure 1, for subsequent analyses.

A certain volume of the pyridine solution was taken while keeping pH, the amount of added composite material, light intensity, temperature and other conditions controlled, and its absorbance was measured after centrifugation after a certain time of reaction. Percentage removal was calculated by converting the standard curve equation to concentration as:

η = [(C_0_ − C_t_)/C_0_] × 100% (1)

where C_0_ and C_t_ are the concentrations of the solution before and after treatment (mg/L), respectively, and η represents percentage removal (%).

### 2.3. Analysis

The crystalline structures of the materials were characterized by the X-ray diffraction (XRD, D8ADVANCE, Bruker, Billerica, MA, USA) method. The surface morphologies of the materials were determined by scanning electron microscopy (SEM, SU 8020, HITACHI, Tokyo, Japan), and their optical properties were evaluated by UV-vis diffuse reflectance spectroscopy (Hitachi, Japan). Amperometric i-t curves were measured on an electrochemical workstation (CHI 660E, CH Instruments, Austin, TX, USA) where the photocurrent light source was provided by a xenon lamp with a cutoff filter.

## 3. Results and Discussion

### 3.1. Characterization Analysis 

**Figure 2:**
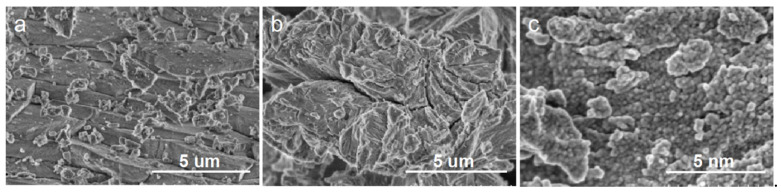
Scanning electron micrographs before and after shell modification and modified loading: font (**a**) SP (5 um), (**b**) MSP (5 um) and (**c**) La-Fe-TiO_2_@ MSP (5 nm).

Scanning electron microscopy images before and after shell modification are shown in Figure 2a,b, respectively. The figure shows that the shells had irregular lamellar structures before and after modification, where the texture of the lamellar layer before modification was tight with no pores. After modification, the surface structure became loose, obvious cavities were observed and a specific surface area was significantly increased, indicating that the modified material had good adsorption ability. La-Fe-TiO_2_@MSP composites were fabricated through the sol-gel method, and their surface morphology is illustrated in Figure 2c. It was seen from the figure that the modified shells were covered with a uniform layer of round spherical TiO_2_ fine particles, indicating good loading, but certain agglomeration phenomena were also observed.

**Figure 3:**
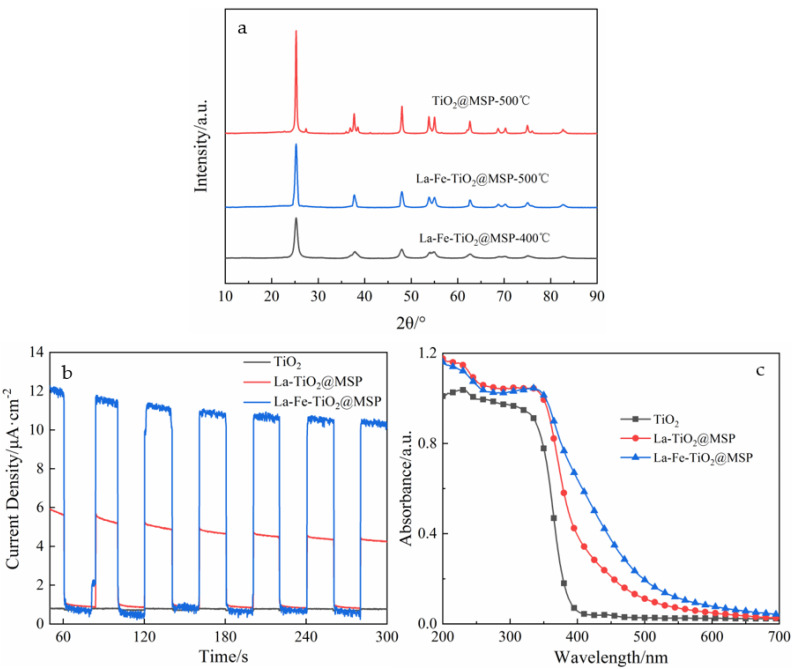
Characterization test charts for different materials: (**a**) XRD patterns of TiO_2_@MSP and La−Fe−TiO_2_@MSP; (**b**) Photoluminescence spectra of TiO_2_, La−TiO_2_@MSP and La−Fe−TiO_2_@MSP; and (**c**) UV−vis diffuse reflectance spectra of TiO_2_, La−TiO_2_@MSP and La−Fe−TiO_2_@MSP.

TiO_2_@MSP and La-Fe-TiO_2_@MSP at different calcination temperatures were prepared, as shown in Figure 3a. The XRD characterization of TiO_2_@MSP at the calcination temperature of 500 °C, La-Fe-TiO_2_@MSP at 400 °C or 500 °C, respectively, showed that the characterization results from the samples all had more distinctive diffraction peaks at about 2θ = 25.26, 37.74, 48.00, 53.84, 55.02 and 62.64 with more obvious characteristic diffraction peaks corresponding to anatase TiO_2_ (101), (004), (200), (105), (211) and (204) crystal plane peaks (JCPSD No. 21-1272), which are all typical of anatase phase crystallization. In addition, when the calcination temperature increased, the intensity of diffraction peaks increased, indicating that the crystallinity of the material was elevated, which was favorable for photodegradation. After this treatment, the peak width became larger, indicating that Ti^4+^ in the lattice was replaced by La^3+^ resulting in lattice distortion, but displacement rate was low, and La^3+^ and Fe^3+^ characteristic diffraction peaks had not appeared, indicating that most of the La^3+^ might be uniformly distributed on the surface of particles in the form of La_2_O_3_ nanoclusters. However, La^3+^ was probably uniformly distributed on the particle surface in the form of La_2_O_3_ nanoclusters without entering its interior. Meanwhile, after entering, Fe^3+^ formed a lattice of Ti-O-Fe with a smaller lattice constant than that of the Ti-O-Ti lattice.

Figure 3b shows photocurrent test graphs for the analysis of the photogenerated carrier separation characteristics of TiO_2_ before and after doping, and its vertical coordinate photocurrent intensity represents the separation efficiency of electron–hole pairs. The transient photocurrent intensities of La-TiO_2_@MSP and La-Fe-TiO_2_@MSP were enhanced by 6 and 14 times, respectively, compared with that of TiO_2_. The doping of La^3+^ and Fe^3+^ could effectively capture the separated electron–hole pairs and reduce the composite of electron–holes. La^3+^ and Fe^3+^ could jointly improve the electron–hole separation efficiency, enhance the transient optical current intensity and the final photocatalytic effect was improved.

Figure 3c illustrates the UV-vis DRS absorption spectra of the three samples of TiO_2_, La-TiO_2_@MSP and La-Fe-TiO_2_@MSP. As was seen from the figure, compared with TiO_2_, the spectral response ranges of La-TiO_2_@MSP and La-Fe-TiO_2_@MSP were more extended, and light absorption capacities in the wavelength range of 400 to 600 nm were significantly enhanced. Additionally, visible absorption capacities were improved, and absorption band edges were red shifted to different degrees. From XRD patterns, it was apparent that the doping of La^3+^ into TiO_2_ inhibited crystal growth, which caused a blue shift in the absorption edge of material, while the doping of La^3+^ generated more oxygen vacancy defects, causing a significant red shift in the absorption edge. UV-vis DRS results clearly showed the overall red shift of absorption band edge, which indicated that La doping enhanced the effects of oxygen vacancy defects and reduced forbidden band widths. With the doping of Fe^3+^, the absorption ability of the sample in the visible range was further enhanced and the red shift of the band edge was increased, due to the reduction of the forbidden band width of TiO_2_ due to the introduction of 2p orbitals by Fe^3+^ doping.

### 3.2. Optimization of Experimental Conditions for Photocatalytic Degradation

#### 3.2.1. Blank Control Group

**Figure 4:**
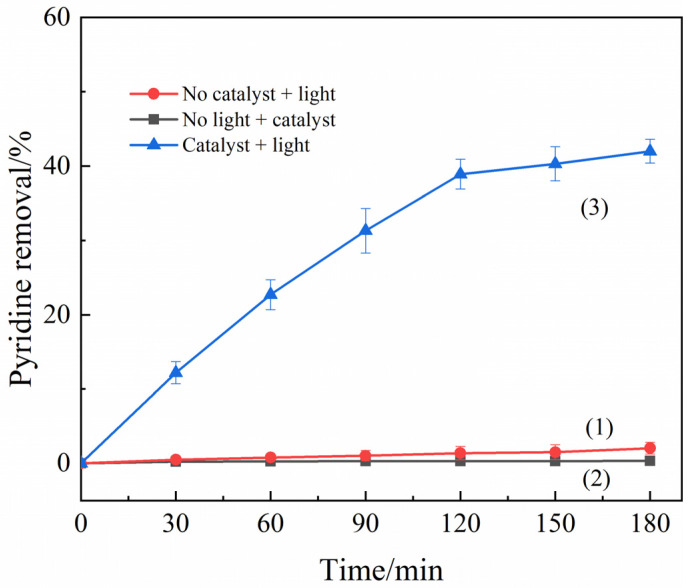
Photocatalytic blank test: (1) no catalyst, light intensity 560 W; (2) no light, photocatalyst dosage 150 mg/L; and (3) photocatalyst dosage 150 mg/L, light intensity 560 W.

To eliminate the interferences of external factors, a blank control group was set up for comparison. Three portions of 100 mL pyridine solution with an initial concentration of 50 mg/L were added to the quartz beaker, and in blank group (1), no catalyst was added and irradiated under 560 W UV light; in blank group (2), 15 mg of photocatalyst was added and treated under dark room conditions; in blank group (3), 15 mg of photocatalyst was added and irradiated under 560 W UV light (Table 1).The obtained results are shown in Figure 4. As is seen in the figure, in blank group (1), the treatment effect did not exceed 2% when the reaction was continued up to 180 min, indicating that only a small amount of pyridine was degraded when only UV light was irradiated without adding the catalyst, and the witnessed effect was negligible. It is the exposure to continuous UV light that induces the production of a small amount of hydroxyl radicals, which in turn leads to photolysis [38]. In blank group (2), there was no degradation rate of pyridine in the presence of catalyst without light, so the interference of composite material adsorption on the photocatalytic degradation of pyridine in the absence of UV irradiation was considered as negligible. The concentrations of pyridine in blank groups (1) and (2) were not significantly changed within 180 min, which combined with the degradation effect of about 45% in control group (3), and it was indicated that no adsorption or degradation of pyridine was observed in the absence of addition or UV light illumination.

#### 3.2.2. Effects of Different Factors on Photocatalytic Degradation 

**Figure 5:**
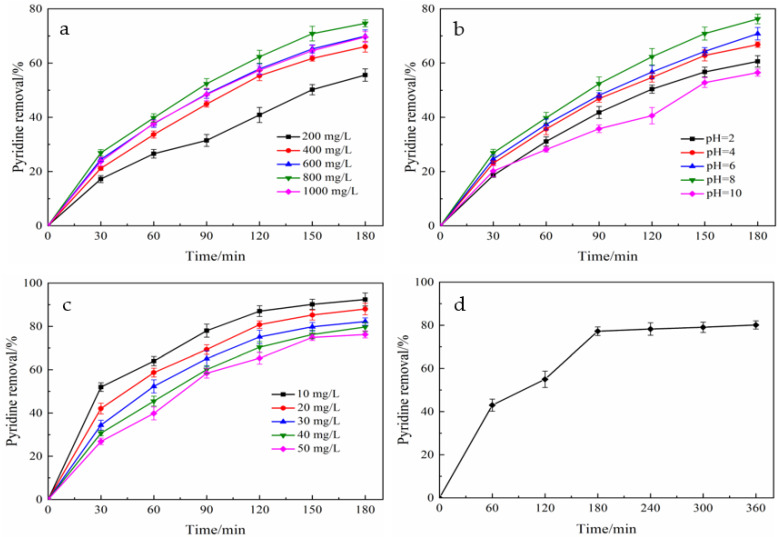
Influencing factors of photocatalytic degradation: (**a**) photocatalyst dosage (initial concentration 50 mg/L, pH = 7, light intensity 560 W, temperature 35 °C, photocatalytic time 3 h); (**b**) pH (initial concentration 50 mg/L, photocatalyst dosage 800 mg/L, light intensity 560 W, temperature 35 °C, photocatalytic time 3 h); (**c**) initial concentration (pH = 8, photocatalyst dosing 800 mg/L, light intensity 560 W, temperature 35 °C, photocatalytic time 3 h); and (**d**) photocatalytic time (initial concentration 50 mg/L, pH = 8, photocatalyst dosing 800 mg/L, light intensity 560 W, temperature 35 °C).

To further validate the photocatalytic removal of pyridine by La-Fe-TiO_2_@MSP and optimize its removal efficiency, the influences of dosage, pH, initial concentration and photocatalytic time on pyridine removal were investigated (Table 1 and Figure 5).

The effect of different doses of La-Fe-TiO_2_@MSP was investigated. The results are illustrated in Figure 5. It is seen from the Figure 5a that when the dosage was 200 mg/L, the pyridine degradation rate after 180 min of reaction was only 55.59%, which was the lowest. However, when the dosage was increased from 400 to 600 mg/L, the pyridine degradation rate increased from 66.04% to 66.96%. It is speculated that the composite photocatalyst was unable to make full use of the photons generated by UV irradiation when the amount of composite material was low during the reaction, and it cannot produce enough strong oxidizing substances such as hydroxyl radicals, resulting in a lower degradation rate [39]. With the increase of the amount of added composite material, the composite catalyst could well utilize UV light to produce more photogenerated electrons e^−^ and holes h^+^, leading to the increase in the degradation rate [40,41]. The strongest effect with the degradation rate of 76.45% was achieved at the dosage of 800 mg/L. When the dosage was further increased to 1000 mg/L, the pyridine degradation rate was decreased. Presumably, when the excessive composite photocatalyst was added, the increase in the catalyst concentration directly led to the aggregation of particles, which produced a masking effect and reduced the effective photon yield. At the same time, an excessive amount of composite photocatalyst resulted in shielding the scattering of light, reducing the transmission depth of UV light resulting in light energy loss, decreasing the utilization of light energy, the production of less oxidants and the reduction in the efficiency of photocatalytic oxidative degradation [41]. 

The influence of pH on the photocatalytic treatment effect of pyridine was examined, and experimental results are illustrated in Figure 5b. It was seen from the graph that the treatment effect was optimal at pH = 8, with the highest degradation rate of up to 71.2%. The key reason was that the adsorption behavior of the catalyst surface was directly influenced by initial pH [41], which meant that pH had a prominent effect on pyridine degradation and the best degradation effect was achieved at neutral pH. Additionally, pH had strong effects on the ability of the catalyst surface to adsorb organic matter, the particle aggregation degree, the number of surface charges and the position of valence band edges and conduction bands [41]. pH not only affected the electrostatic interactions between photocatalyst and target removal, but also had a great influence on the binding of holes and hydroxyl groups on the photocatalyst surface and the formation of reaction products [42,43]. pH was low when weakly basic pyridine was protonated and partially positively charged, which was not conducive to surface adsorption on the catalyst. Moreover, acidic conditions reduced the generation of hydroxide radicals by holes and OH^−,^,which also reduced the ability to degrade organic matter. However, at higher pH values, the surface adsorption of the catalyst was reduced due to the negative charge of the material surface, which was unfavorable to capture photogenerated electrons (e^−^), resulting in a significant reduction in photocatalytic capacity.

The effect of the initial solution concentration on the photocatalytic treatment effect of pyridine was also evaluated, and the experimental results are illustrated in Figure 5c. With the increase in the initial concentration of the pyridine solution, photocatalytic degradation efficiency was gradually decreased. As can be seen from Figure 5c, when the initial pyridine solution concentration was 10 mg/L, the photocatalytic degradation rate was the highest (90.10%) after continuous reaction for 180 min. As the initial concentration was increased to 50 mg/L, the photocatalytic degradation rate was the lowest after reaction for 180 min, which was only 76.25%. It was speculated that this was because of the fact that at low concentrations, the amount of pyridine adsorbed by the photocatalyst was relatively low; and the reaction mainly occurred among active sites, oxygen molecules and water molecules; and pyridine was oxidized by active radicals [36]. When the concentration was gradually increased, the amount of adsorption was increased and under light conditions, the active sites of the catalyst generated electron–hole pairs, which oxidatively reduced the pyridine molecules adsorbed on the catalyst surface, leading to a reduced final degradation rate.

Bahnemann et al. [44] concluded that the initial concentration of the target pollutant mainly influenced its adsorption rate and the intermediate products formed by its degradation on photocatalyst surface active sites. For wastewaters with high initial concentrations, the removal of organic substances was mainly achieved through the adsorption onto the photocatalyst surface followed by electron–hole oxidation. Furthermore, with the increase in the amount of adsorbed organic substances, the number of active sites on the surface occupied after adsorption was increased, so that the removal effect of photocatalysis on the total organic substances was enhanced. In the reaction stage, the content of intermediates was increased with the concentration of reactants, resulting in the adsorption of a large number of intermediates on the photocatalyst surface and competition for surface active sites. At the same time, the high concentration of reactants decreased light penetration, the number of effective photons and the reaction rate [43]. At lower initial concentrations of solution, the reaction process was faster, but the removal amount was limited, and economic issues had to be further considered to select the appropriate initial concentration in practical applications.

Figure 5d shows the effects of different photocatalytic reaction times on the photocatalytic degradation of pyridine, and it is clearly seen from the figure that the pyridine degradation rate presented a clear increasing trend with time and the degradation rate of photocatalytic reaction was about 81% at 3 h. Meanwhile, the increase rate of the degradation rate was gradually decreased as the reaction continued. From the perspective of energy saving, the photocatalytic time could be set at 3 h.

#### 3.2.3. Effect of Coexisting Substances in Water on Degradation of Pyridine

**Figure 6:**
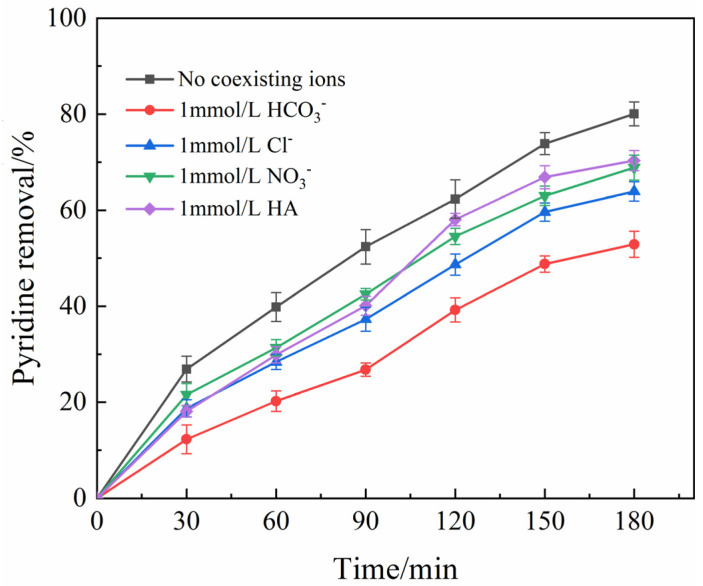
Effect of coexisting ions on the degradation of pyridine (pH = 8, La−Fe−TiO_2_@MSP dosing 800 mg/L, pyridine initial concentration 50 mg/L, photocatalytic time 3 h, light intensity 560 W, temperature 35 °C).

In actual natural water bodies, there may be a large number of anions and natural organic compounds, which will affect the degradation efficiency of pollutants by depleting the active substances in the reaction system. The effects of 1.0 mmol/L Cl^−^, HCO_3_^−^ and NO_3_^−^ and humic acid (HA) on the photocatalytic oxidation of pyridine by La-Fe-TiO_2_@MSP were investigated, and the results are shown in Figure 6. Both anions and humic acids in the water column inhibited photocatalysis, and the inhibition effect of HCO_3_^−^ was the most obvious, mainly because HCO_3_^−^ had a stronger scavenging effect on ·OH, which had a greater impact on the photocatalytic effect compared with the capture of ·OH by Cl^−^. In addition, under UV irradiation, NO_3_^−^ acted as an inert filter layer, but La^3+^ could increase the catalytic surface charge and promote the adsorption of anions, and was less hindering than other anions.

### 3.3. Kinetic Analysis

The photocatalytic degradation rate refers to how fast the concentration of a substrate changes with time during the photocatalytic degradation process. By studying the catalyst degradation rate, the catalytic activity of the catalyst could be further evaluated according to the degradation rate index method.

The Langmuir–Hinshelwood Kinetic Equation, as shown below, could be used for all photocatalytic oxidation reactions to determine the degradation rates of organic matter by catalysts during photocatalytic degradation:

−ln(C/C_0_) = kt (2)

where C and C_0_ are post-reaction and initial concentrations, respectively, and k is slope, indicating reaction rate constant.

The ln(C/C_0_)-t curve was linear, and the photocatalytic reaction behaved as a primary reaction. To investigate the kinetic mechanism of pyridine photocatalytic degradation by La-Fe-TiO_2_@MSP, the obtained experimental data were fitted according to the kinetic equation(s) and the fitted models were compared according to reaction rate constants (k) and correlation coefficients (R^2^).

**Figure 7:**
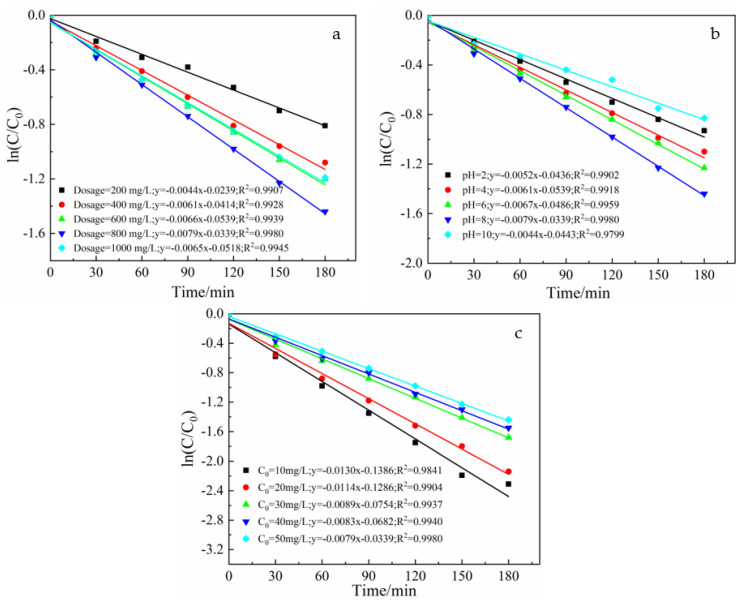
ln(C/C_0_)−t relationship curve for different variables: (**a**) composite photocatalyst dosage, (**b**) different pH values and (**c**) different concentration.

As shown in Figure 7a, the correlation coefficient of ln(C/C_0_)-t for composite photocatalyst dosage was close to 1 (Table 1), showing a linear correlation, and the process of photocatalytic degradation at different dosage levels could be described by first-order kinetic equation. The photocatalytic degradation rate reached the maximum value of 0.0079 h^−1^ for the compound photocatalytic material dose of 800 mg/L. Subsequent experiments revealed that compound photocatalysts were all dosed at 800 mg/L.

Combining Figure 7b and Table 1, it can be seen that the ln(C/C_0_)-t relationship curves for different pH conditions showed a linear correlation with the correlation coefficient of close to 1 (the specific values are shown in Table 1), and photocatalytic degradation reactions under different pH conditions could be described as first-order kinetic equations. The photocatalytic degradation rate reached the maximum value of 0.0079 h^−1^ at pH = 8, and the solution pH for subsequent experiments was set at 8.

Combining Figure 7c and Table 1, it can be seen that reaction rate constant K tended to decrease with the gradual increase in the initial concentration of the solution. The increase in concentration increased the amount of organic matter or intermediates adsorbed onto the surface and photocatalytic consumption of active radicals. Keeping other reaction conditions constant, the relative lack of active radicals directly decreased the degradation rate and rate constant [43]. At the same time, the increase in concentration increased the yield of intermediates, which were all adsorbed onto the photocatalyst surface [44]. These intermediates were diffused more slowly, causing the deactivation of active sites on the surface and decreasing the photocatalytic effect [45]. The ln(C/C_0_)-t relationship curves for different initial concentrations showed a linear correlation with correlation coefficients of close to 1 (Table 1) and photocatalytic degradation reactions under different initial concentrations were described by first-order kinetic equation. In practical applications, a suitable initial concentration should be selected considering economic efficiency and operational simplicity.

### 3.4. Comparative Study of Different Materials

To demonstrate the excellent photocatalytic performance of La-Fe-TiO_2_@MSP and its development potential, the pyridine photocatalytic degradation by five proposed materials of TiO_2_, Fe-TiO_2_, La-TiO_2_, La-Fe-TiO_2_ and La-Fe-TiO_2_@MSP under the same conditions was investigated.

**Figure 8:**
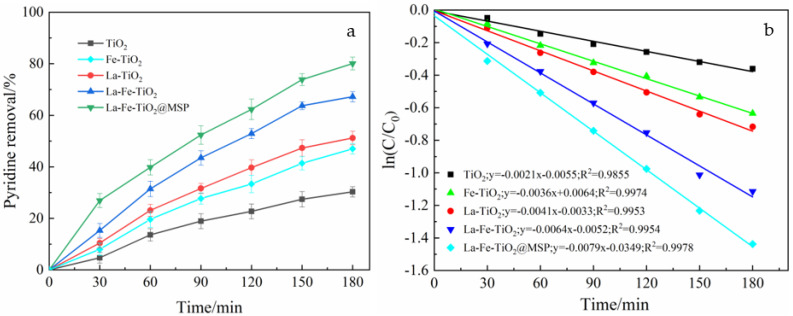
Comparison of degradation effects of different types of photocatalysts and ln(C/C_0_)−t relationship curve: (**a**) Effect of five photocatalytic materials on pyridine removal under the same conditions, (**b**) ln(C/C_0_)−t relationship curves for five different materials under the same conditions (TiO_2_, Fe−TiO_2_, La−TiO_2_, La−Fe−TiO_2_ and La−Fe−TiO_2_@MSP).

Equal amounts of pyridine solution with an initial concentration of 50 mg/L and pH = 8 were prepared. During the tests, light intensity was controlled at 560 W, temperature was fixed at 35 °C and equal amounts of 800 mg/L TiO_2_, Fe-TiO_2_, La-TiO_2_, La-Fe-TiO_2_ and La-Fe-TiO_2_@MSP were added to the solutions and their absorbance was measured for 180 min to investigate the effects of different catalysts on photocatalytic degradation (Table 1). The results of the degradation experiments are shown in Figure 8.

From Figure 8a, it is seen that the pyridine photocatalytic degradation by La-Fe-TiO_2_@MSP was significantly better than those by the other four materials. According to the experimental data of pyridine photocatalytic degradation kinetics by the above five materials, the curves obtained by a linear fitting of ln(C/C_0_)-t are shown in Figure 8b, and the solved primary reaction rate constants are summarized in Table 1. The results showed that the rate constant of the photocatalytic degradation of pyridine by La-Fe-TiO_2_@MSP was the highest (0.0079 h^−1^) under the same experimental conditions.

In other words, the ln(C/C_0_)-t relationship curves of the five materials showed a linear correlation with a correlation coefficient of close to 1. A first-order kinetic equation was used to describe the photocatalytic degradation reactions of different catalysts. La-Fe-TiO_2_ /MSP had the highest rate constant, the best catalytic performance and the highest development potential.

### 3.5. Photocatalytic Degradation Mechanism

#### Quenching Experiment

**Figure 9:**
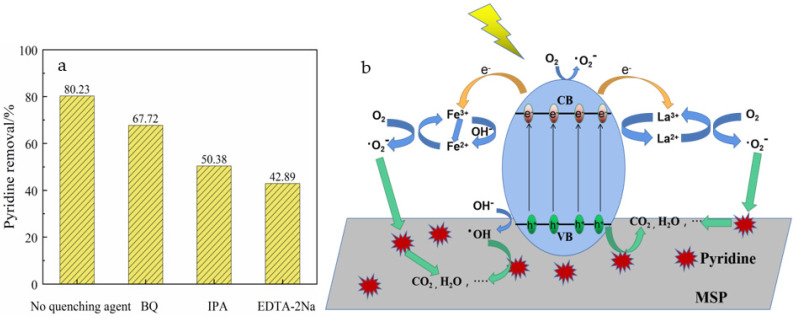
(**a**) Effects of different scavengers on photocatalytic degradation of pyridine and (**b**) proposed mechanism for pyridine photodegradation process using La−Fe−TiO_2_@MSP.

To determine the types of active substances that made a difference in the reaction of La-Fe-TiO_2_@MSP, the quenching experiments were carried out under optimum reaction circumstances. The quenching experiments aimed to accomplish the capture of hydroxyl radicals, hole and superoxide radicals by adding a certain amount of isopropanol (IPA), disodium ethylene diamine tetraacetate (EDTA-2Na) and p-benzoquinone (BQ), respectively. The experimental results are shown in Figure 9a. The effect of the addition of EDTA-2Na on the degradation of pyridine indicated that the reactive groups generated by the reaction of photogenerated electrons with h+ were the main reactive species for the degradation of pyridine. Furthermore, the addition of BQ and IPA also reduced the degradation rate of pyridine by 12.51% and 29.85%, respectively. This phenomenon may be caused by the decrease in hole consumption and increase in electron–hole complexation rate after the quenching of superoxide radicals and hydroxyl radicals, and also indicates that both superoxide radicals and hydroxyl radicals play an oxidizing role in the degradation process.

TiO_2_ + hv → h^+^_VB_ + e^−^_CB_ (3)

H_2_O + h^+^ → OH + H^+^ (4)

O_2_ + e^−^ → O_2_^−^ (5)

H^+^+·O_2_^−^ →·OOH (6)

OOH + OOH + e^−^ →·OH + OH^−^ + O_2_ (7)

Pyridine + (OH,·O_2_^−^, h^+^) → CO_2_ + H_2_O (8)

Figure 9b shows the mechanism of photocatalytic degradation of pyridine by La-Fe-TiO_2_@MSP. After the La-Fe-TiO_2_@MSP surface was irradiated by ultraviolet light, an electron–hole pair (Equation (3)) was generated, the hole reacted with OH^−^ in H_2_O to generate hydroxyl radical (OH) (Equation (4)) and the electron reacted with O_2_ to generate superoxide radical anion (O_2_^−^) (Equation (5)). O_2_^−^ and OH were active substances, and the hole (h+) and the above two active substances could react with pyridine to mineralize it and finally form CO_2_ and H_2_O (Equations (6)–(8)). By doping La into La3d hybridization orbital, La^3+^ increased the surface defects of TiO_2_ lattice, making it capable of effectively capturing the separated electron–hole pairs, and enhancing TiO_2_ surface carriers and photocatalytic performance. Doped Fe^3+^ introduced Fe2p hybrid orbital and Fe^3+^ with hydroxide ions forming strong oxidizing hydroxyl radicals under light irradiation. Finally, La^3+^ and Fe^3+^ co-doping generated a synergistic effect to capture the separated electron–hole pairs. The doping of La and Fe reduced the band gap energy of titanium dioxide and improved the photocatalytic degradation effect. In addition, MSP was an excellent carrier to promote the photodegradation process by adsorbing pyridine. After MSP adsorption, pyridine molecules were transferred to TiO_2_ particles through the interface, thus forming a complete adsorption photodegradation process.

## 4. Conclusions

The La-Fe-TiO_2_@MSP composite photocatalyst was prepared by the sol-gel method and evaluated by the SEM analysis. The modified shell was covered with a layer of uniform round spherical TiO_2_ fine particles with good loading. Clear diffraction peaks were observed on XRD patterns, all of which were characteristic of the anatase phase. UV-vis DRS showed that the spectral response range of La-Fe-TiO_2_@MSP was extended and light absorption capacity was significantly increased from 400 to 600 nm. Additionally, the visible light absorption capacity was enhanced, and the absorption band edge was red shifted to different degrees. Photocurrent tests revealed an increase in photogenerated carriers and electron–hole separation efficiency. The results of the photodegradation experiments showed that the maximum removal rate of 80.23% was achieved by controlling pH = 8, temperature 35 °C, light intensity 560 W, 800 mg/L catalyst added to 50 mg/L pyridine solution and photocatalytic reaction for 180 min. Kinetic studies yielded the degradation process of La-Fe-TiO_2_@MSP conformed to the primary reaction kinetic equation, and the degradation rate constant under optimal reaction conditions was 0.0079 h^−1^, with the best catalytic degradation performance compared with the other four materials. The quenching experiment determined that the active species in the photodegradation process was dominated by holes and hydroxyl radicals, which can be mineralized to achieve efficient removal, further resolving water resources and environmental conflicts and promoting clean production and green development of the coal chemical industry.

## Figures and Tables

**Table 1:** List of experimental reaction control conditions (temperature 35 °C).

Figure	Group	Dosage (mg/L)	pH	Initial Concentration (mg/L)	Photocatalytic Time (h)	Light Intensity (W)	k (h^−1^)	R^2^
4	1	—	9	50	3	560	—	—
	2	150				—	—	—
	3	150				560	—	—
5	a	200	7	50	3	560	0.0044	0.9907
		400					0.0061	0.9928
		600					0.0066	0.9945
		800					0.0079	0.9939
		1000					0.0065	0.9980
	b	800	2	50	3	560	0.0052	0.9902
			4				0.0061	0.9918
			6				0.0067	0.9959
			8				0.0079	0.9980
			10				0.0044	0.9799
	c	800	8	10	3	560	0.0130	0.9841
				20			0.0114	0.9904
				30			0.0089	0.9937
				40			0.0083	0.9940
				50			0.0079	0.9980
	d	800	8	50	1	560	—	—
					2		—	—
					3		—	—
					4		—	—
					5		—	—
					6		—	—
7	1	800	8	50	3	560	0.0021	0.9855
	2						0.0036	0.9974
	3						0.0041	0.9953
	4						0.0064	0.9954
	5						0.0079	0.9978
